# Projected increases in surface melt and ice loss for the Northern and Southern Patagonian Icefields

**DOI:** 10.1038/s41598-021-95725-w

**Published:** 2021-08-19

**Authors:** Claudio Bravo, Deniz Bozkurt, Andrew N. Ross, Duncan J. Quincey

**Affiliations:** 1 Centro de Estudios Científicos (CECs), Valdivia, Chile; 2 School of Geography, University of Leeds, Leeds, UK; 3 Department of Meteorology, University of Valparaíso, Valparaíso, Chile; 4 Center for Climate and Resilience Research (CR)2, Santiago, Chile; 5 School of Earth and Environment, University of Leeds, Leeds, UK

**Keywords:** Cryospheric science, Projection and prediction

## Abstract

The Northern Patagonian Icefield (NPI) and the Southern Patagonian Icefield (SPI) have increased their ice mass loss in recent decades. In view of the impacts of glacier shrinkage in Patagonia, an assessment of the potential future surface mass balance (SMB) of the icefields is critical. We seek to provide this assessment by modelling the SMB between 1976 and 2050 for both icefields, using regional climate model data (RegCM4.6) and a range of emission scenarios. For the NPI, reductions between 1.5 m w.e. (RCP2.6) and 1.9 m w.e. (RCP8.5) were estimated in the mean SMB during the period 2005–2050 compared to the historical period (1976–2005). For the SPI, the estimated reductions were between 1.1 m w.e. (RCP2.6) and 1.5 m w.e. (RCP8.5). Recently frontal ablation estimates suggest that mean SMB in the SPI is positively biased by 1.5 m w.e., probably due to accumulation overestimation. If it is assumed that frontal ablation rates of the recent past will continue, ice loss and sea-level rise contribution will increase. The trend towards lower SMB is mostly explained by an increase in surface melt. Positive ice loss feedbacks linked to increasing in meltwater availability are expected for calving glaciers.

## Introduction

**Figure 1:**
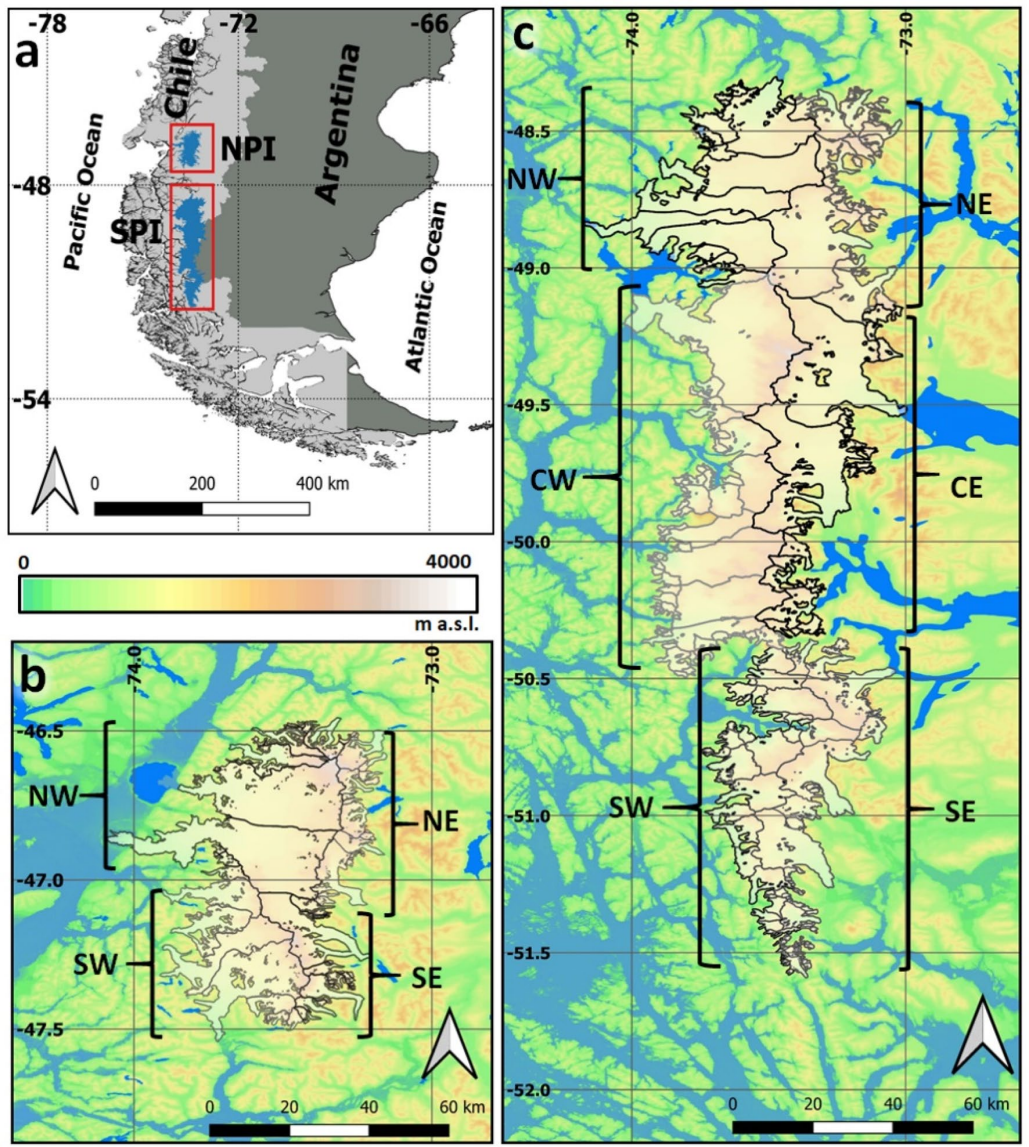
Location of the NPI and SPI in Southern South America (**a**). Details of the NPI (**b**) and the SPI (**c**). Colour shading is the topography from SRTM at 90 m spatial resolution and blue tones are lakes and fjords. In the NPI the main glaciers per each sub-zone are: San Rafael and San Quintin (NW), Exploradores, Soler and Nef (NE), Steffen and Acodado (SW) and Colonia, Pared Norte and Pared Sur (SE). In the SPI: Jorge Montt, Témpano, Bernardo, Occidental and Greve (NW), O’Higgins and Chico (NE), Pio XI, HPS12, HPS13, Europa and Guilardi (CW), Viedma and Upsala(CE), Calvo, Asia and Amalia (SW) and Perito Moreno, Grey and Tyndall (SE). Maps (**a**–**c**) were made using QGIS 3.12 București (www.qgis.org).

Meltwater generated by receding land ice is impacting global sea-level at an accelerating rate^1^. The glaciers of the Southern Andes represent one of the highest contributors, responsible for a total of 3.3 mm of sea-level rise between 1961 and 2016^2^. This region includes two large temperate icefields: the Northern Patagonian Icefield (NPI) and the Southern Patagonian Icefield (SPI) (Fig. 1). In recent years, both icefields have been the focus for studies of geodetic mass balance^3–12^. These analyses are uniform in their assessment, indicating that overall ice mass loss has increased through time for both Patagonian Icefields, although patterns on the SPI are somewhat heterogeneous^9,12^. Together, the Patagonian Icefields represent 83% of the total ice loss in the Southern Andes^10^ and it has been estimated that the sea-level contribution of Patagonian glaciers in the last 50 years is one order of magnitude larger than at the Little Ice Age (LIA) maximum, dated AD1650 in the SPI and AD1870 in the NPI^13^.

The primary conditions shaping the response of Patagonian glaciers are complex^14^, defined at a broad-scale by the interaction of the Andean topography with the dominant westerlies and water vapour flux transported at these latitudes, which creates a strong climatic divide^15^, particularly in terms of humidity and precipitation^16^. West–east spatial differences in local (icefield-scale) climate characteristics have been described in terms of cloud cover^17^, air temperature lapse rates, glacier cooling effect^18^, snow accumulation^19^ and the occurrence of föhn events^20^. In turn, these characteristics define west–east differences over the glacier surface, for instance, in producing variable snow facies^21^ and leading to asynchronous melt seasons^22^.

There is a discrepancy between positively modelled surface mass balances using climate data^23–26^ and negative total mass balances derived by the geodetic approach. It has been suggested that the main cause of this discrepancy is that climate models which are used to force SMB^23–26^ tend to overestimate the precipitation rate in Patagonia^15^, leading to the positive SMB results. Furthermore, the discrepancy can also be partly explained by the importance of the frontal ablation mechanisms in calving glaciers. Most Patagonian Icefield glaciers terminate in lacustrine or marine calving settings. In the case of the NPI, the frontal areas mostly terminate in relatively small and shallow lakes, while the SPI glaciers terminate in fjords on the west side and deep and large lakes on the east side. Frontal ablation ice loss is associated with adjustments to the ice dynamics of large glaciers^10^ and/or the lake/fjord characteristics of each glacier^27^ but is not typically included in SMB.

Climate conditions that define the broad-scale surface mass balance (SMB) also play a primary role in driving glacier-scale ablation. Fundamentally, climate conditions define the energy available for melt. Air temperature is important, but turbulent fluxes are high in Patagonia, which in turn depend on wind speed, humidity gradients and near-surface air temperature, among others^28,29^. Secondly, feedbacks between ice dynamics and SMB have been identified for the glaciers in this region. Patagonian glaciers comprise temperate ice^30^ leading to the presence of abundant meltwater, enhancing crevassing in the terminus areas and leading to flotation and increasing frontal ablation^31^. Additionally, a strong correlation between ice speed and air temperature fluctuations has been evidenced for the calving terminus of the Perito Moreno Glacier in the SPI^32,33^, suggesting that the meltwater infiltrating into the glacier increases basal water pressure, which in turn increases basal sliding and thus overall ice speed.

In view of the local, regional and worldwide impacts observed due to the ongoing glacier shirnkage of Patagonian glaciers, and the direct and indirect importance of the climate conditions that drive the total ice loss, an assessment of the potential future SMB is both critical and timely. The aim of this work is therefore to estimate the SMB and its components, including the surface ablation, accumulation and sublimation for both the NPI and the SPI (Fig. 1) until the year 2050.

We used dynamically downscaled outputs of the MPI-ESM-MR Earth System Model^34^ obtained with Regional Model RegCM4.6 at 10-km spatial resolution and daily time step for the historical period^35^ (hydrological years April–March between 1976/77 and 2004/05) and for two Representative Concentration Pathways (RCP2.6 and RCP8.5, between hydrological years 2005/06 and 2049/50). The choice of the MPI-ESM-MR Earth System Model was made because it is one of the best available for representing the large-scale climate characteristics of the Southeast Pacific^35^. RCPs were chosen to best represent current emissions (RCP8.5)^36^, and to best represent temperature targets as laid out by the 2015 Paris Agreement (RCP2.6)^37^. These products were used directly to estimate snow accumulation and its changes, and subsequently to feed an energy balance model (EBM) to estimate the ablation and its changes over both Patagonian Icefields. Changes in the equilibrium-line altitudes (ELA) obtained from the SMB were also analysed and first-order estimations of the ice loss and its associated contribution to sea-level rise until 2050 were derived.

This approach represents a first assessment of the potential future response of the Patagonian Icefields glaciers using daily data until 2050. The focus here is to assess how these icefields are responding to ongoing climate change and what their trajectories will be given future climate scenarios. In the first section of this manuscript, we compare the results of the historical SMB run with previous estimations of modelled and geodetic mass balance, as well as the ELA, at the scale of the two icefields. Then, we present the results of the future SMB and ELA under both scenarios and we quantify the changes in comparison to the historical period as well as estimating total ice loss. Finally, we discuss the implications of the results in terms of potential glacier response and feedbacks associated with changes in ELA, SMB and their components.

## Results

### Historical SMB and ELA

**Figure 2﻿:**
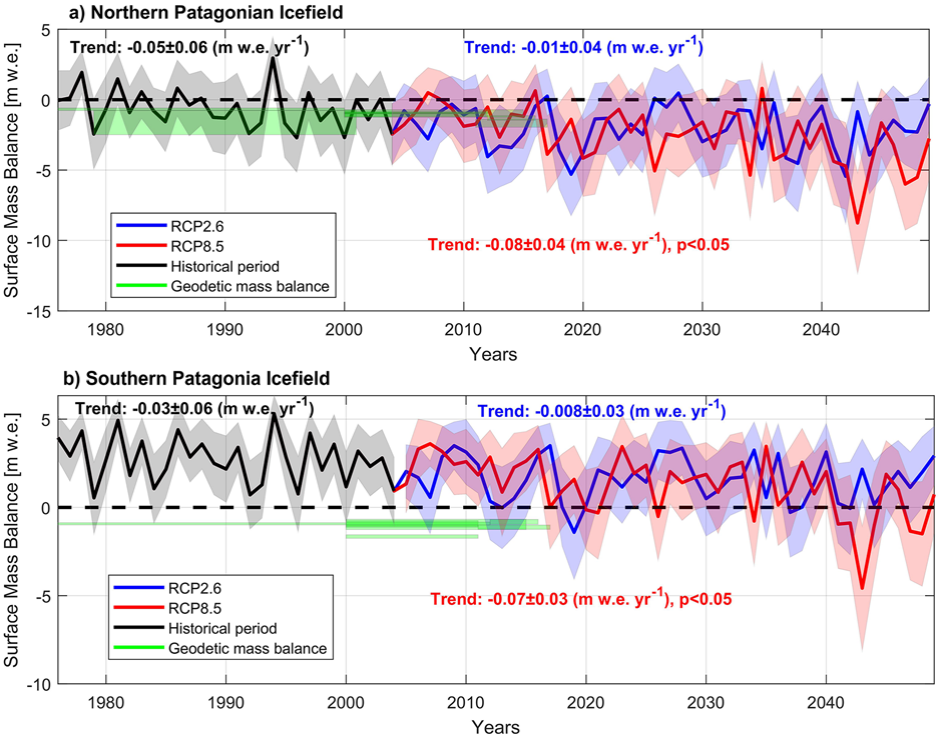
Time series of the modelled surface annual mass balance. (**a**) NPI and (**b**) SPI. Colour shadow represent the uncertainties in the modelled SMB for the historical period and the two scenarios. This shadow area is due to the parametrization of the glacier cooling effect and the method used for the phase partitioning in the total precipitation to define the surface snow accumulation. Green coloured bars indicate previously derived geodetic mass balances and their error range previously estimated in NPI^3,4,6,8–12^ and in the SPI^3,5,7,9–12^. Trends with the 95% confidence interval are shown. Significant trends are also indicated when p < 0.05.

For the historical period, the mean SMB was negative in the NPI, reaching an annual mean value of − 0.6 ± 2.1 m w.e. yr^−1^, in agreement with previously published geodetic mass balances in comparable periods^3,4,6,8–12^ (Fig. 2a, Table S2) and with previously modelled SMBs (e.g. mean of − 0.2 ± 0.7 m w.e. yr^−1^ for the period 1979/80–2009/10^23^). One previous study^26^ suggested a slightly positive modelled SMB of 0.2 ± 0.5 m w.e. yr^−1^ for the period 1979/80–2013/14, but this is not inconsistent with our computation given the relative large uncertainty. This can be partly explained by noting that the periods of analysis are not directly comparable in each case. For the common period between the hydrological years of 1979/80 and 2003/04, we obtained an annual mean of − 0.7 ± 2.1 m w.e. yr^−1^ while Mernild et al.^26^ estimated 0.1 ± 0.5 m w.e. yr^−1^. Additionally, we note that all previous values are in the uncertainty range estimated for our historical SMB, which is higher than previous assessments and reflects the uncertainty mainly associated with snow accumulation and the parametrization of the glacier cooling effect (see Data and Methods section).

In the SPI (Fig. 2b), SMB was positive with a mean of 2.7 ± 1.6 m w.e. yr^−1^ during the historical period, in agreement with the positive SMB computed by previous modelling studies, although our results exceed those of 1.8 ± 0.4 m w.e. yr^−1^^26^ and 2.3 ± 0.9 m w.e. yr^−1^^25^ between 1979/80 and 2009/10. For the hydrological years between 1979/80 and 2003/04 we obtained 2.7 ± 1.6 yr^−1^ m w.e. and Mernild et al.^26^ derived a value of 1.7 ± 0.4 yr^−1^ m w.e. Geodetic mass balances were all negative^3,5,7,9–12^ and outside of our uncertainty range (Fig. 2b, Table S3). There was a slightly decreasing trend during the historical period in the SMB which was not significant, but which contradicts previous SMBs for a similar period that showed a positive trend^24,25^.

The mean long-term ELA was estimated for the historical period for each sub-zone defined in Fig. 1. Overall, the west side showed a lower ELA in comparison to the east side glaciers for both icefields. Although there are no previous estimations that can be directly compared with our ELAs (as most of them are for particular years), observing the end-of-summer snowline using satellite images^4,5,38–40^ provides some form of validation (Table 1). Our modelled values compared to those observed in particular years, tend to overestimate the ELA in the eastern side of both icefields, and notable differences are found along the western margins, where our ELA was generally 100–300 m lower than previous estimations depending on the parameterization (e.g. cooling effect).

**Table 1:** Compa﻿rison of the ELA per sub-zone obtained in this work for the historical period with those obtained by observations of the end of summer snowline using satellite images^4,38–40^ and by SMB modelling^23,25^.

a) NPI	Aniya^38^ (Direct observation 1983–1986 and aerial photographs)	This work for the same years of Aniya^38^	Rivera et al.^4^ (ASTER 10/Feb/2002)	This work for the same year of Rivera et al.^4^	Barcaza et al.^39^ (Landsat between 1979 and 2003)	This work for the same years of Barcaza et al.^39^	Schaefer et al.^23^ (SMB modelling 1975–2011)	This work 1975–2005
NW	1031	849 ± 201	1019	756 ± 176	949 ± 41	835 ± 207	1185 ± 6	792 ± 187
NE	1217	1491 ± 107	1224	1446 ± 156	1371 ± 154	1462 ± 136	1387 ± 4	1433 ± 155
SW	1044	990 ± 117	1026	968 ± 145	924 ± 192	1018 ± 147	1163 ± 6	971 ± 134
SE	1075	1415 ± 66	1138	1407 ± 77	1124 ± 101	1414 ± 68	1351 ± 7	1396 ± 86

**b) SPI**	**De Angelis**^39^** (MODIS Feb. 2002 and Feb. and March 2004)**		**This work for the same years De Angelis**^39^		**Schaefer et al.**^25^** (SMB modelling 1975–2011)**		**This work 1975–2005**	
NW	928 ± 44		728 ± 93		1112		684 ± 98	
NE	1164 ± 38		1335 ± 171		1282		1232 ± 247	
CW	1017 ± 53		703 ± 100		989		669 ± 92	
CE	1248 ± 55		1410 ± 127		1249		1351 ± 153	
SW	1042 ± 62		656 ± 80		900		632 ± 72	
SE	1018 ± 28		1103 ± 118		1023		1048 ± 152	

Previous values are the mean of several glaciers observed. Number of glaciers are variable depending availability in the references. The uncertainty range in our work is given by the sensitivity of the model to the parametrization of the cooling effects and the methods used to compute the partitioning of rain and snow.

A disagreement between the end-of-summer snowline and the mean long-term ELA along the western margins is likely to reflect the high mass turnover observed in maritime glaciers. In addition, snow accumulation events also occurs in summer months^19^, but a high post-event 0 °C isotherm means much of this snow is quickly lost to melt^39^. Additionally, high uncertainty in the snow accumulation rates could lead to a high uncertainty of our modelled ELA. The underestimation in ELA values compared to end-of-summer snowlines suggests that overestimation in the snow accumulation rate could be the main cause of this discrepancy.

### Projected changes in SMB and ELAs

Projected SMB results (which as stated do not account for frontal ablation mechanisms) indicate that the NPI will be characterized by a notable negative SMB until at least 2050. Solely driven by climate forcing, the SPI will continue to gain mass but with lower values compared to the historical period (Fig. 2). In the NPI, the mean projected SMB values were − 2.0 ± 2.5 m w.e. yr^−1^ (RCP2.6) and − 2.5 ± 2.6 m w.e. yr^−1^ (RCP8.5), while in the SPI, the projected mean SMB values were 1.6 ± 2.0 m w.e. yr^−1^ (RCP2.6) and 1.3 ± 2.1 m w.e. yr^−1^ (RCP8.5). However, after the year 2040, a predominance of negative annual SMB in the SPI is projected.

There are notable differences in the interannual variability in the SMB between RCPs. For instance, some particular years from the RCP2.6 scenario were lower in magnitude than those arising from the RCP8.5 SMB run, indicating that the EBM used here seems to capture feedbacks related to other meteorological variables used to feed the model. This is important as it has been demonstrated that the surface ablation of the Patagonian glaciers depends to a large extent on turbulent fluxes, especially the sensible heat flux^28,29^, which in turn also depends on the wind speed. However, the natural interdecadal variability also could play a role in this behaviour, especially over the first decades of the projection. After 2040, the differences between both scenarios are greater as the higher signal-to-noise ratio for the RCP8.5 scenario dominates (as it has a more prominent temperature increase). As might be expected, long-term mean values show that RCP8.5 yielded the greatest reductions in SMB for both icefields.

**Figure 3:**
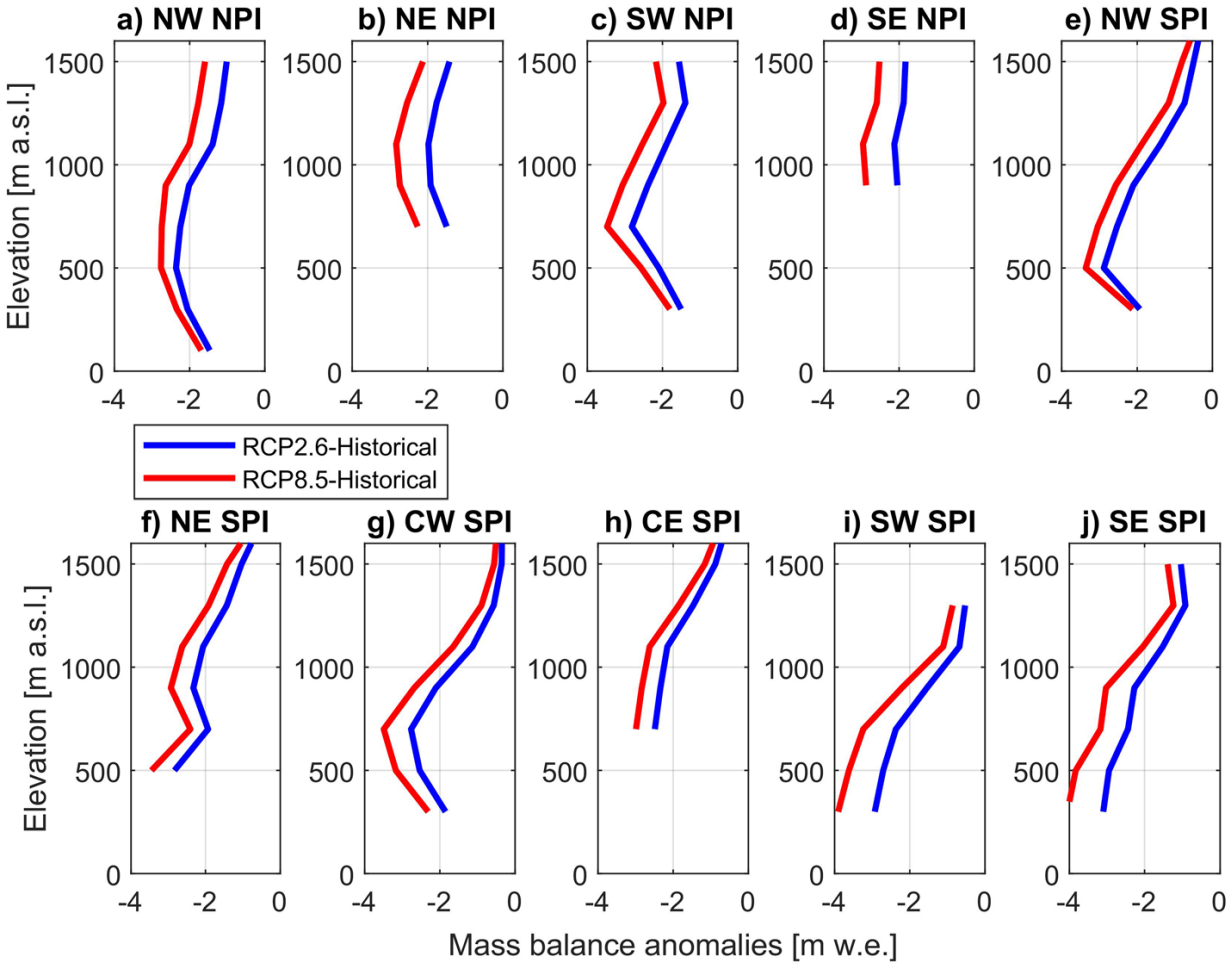
Surface mass balance differences per sub-zone (Fig. 1) and elevation range in both icefields. The differences were derived by comparing long-term SMBs between the historical period and each RCP scenario.

Figure 3 shows the projected mean SMB differences compared to the historical period per elevation range, for each RCPs and for the different sub-zones defined for both icefields (Fig. 1). The reductions in SMB were homogeneous within different regions, but with some differences in the magnitude between regions, being notably larger in the lower section of the south of the SPI (Fig. 3i,j) and lower in the higher elevations (i.e. plateau zone) of the north-central section of the SPI (Fig. 3e,f,g,h). Due to the RegCM4.6 resolution, the higher zones at each icefield (> ~ 1500 m a.s.l.) are not represented well (see Fig. S1).

From the SMB profiles (see the Equilibrium-Line Altitude Methods section), we estimated the mean long-term ELA for each sub-zone and the two pathway scenarios (2005/06–2949-50). Depending on the scenario and the SMB uncertainties, the magnitude of the ELA increase compared to the historical period could reach 118 ± 42 m in the NPI, while in the SPI could reach a maximum of 94 ± 73 m (Table 2).

**Table 2:** Range of difference in the mean long-term ELA per sub-zone between historical period and each RCP scenario.

Icefield	Sub-zone	RCP2.6	RCP8.5
NPI	NW	92 ± 35	118 ± 42
	NE	49 ± 39	62 ± 50
	SW	70 ± 24	86 ± 29
	SE	26 ± 36	30 ± 40
SPI	NW	49 ± 9	66 ± 16
	NE	82 ± 65	94 ± 73
	CW	45 ± 18	58 ± 21
	CE	49 ± 31	55 ± 31
	SW	36 ± 10	49 ± 15
	SE	57 ± 22	69 ± 25

ELA was obtained as the long-term mean of the elevation where the SMB reached 0 m w.e. The range of difference at each scenario is given by the runs using − 1 °C and − 3 °C of cooling effect as meteorological observations suggests^18^ as well as the uncertainty given by the different methods used to the partitioning between rain and snow^19^.

### Annual time series and trends in accumulation, melt and sublimation

**Figure 4:**
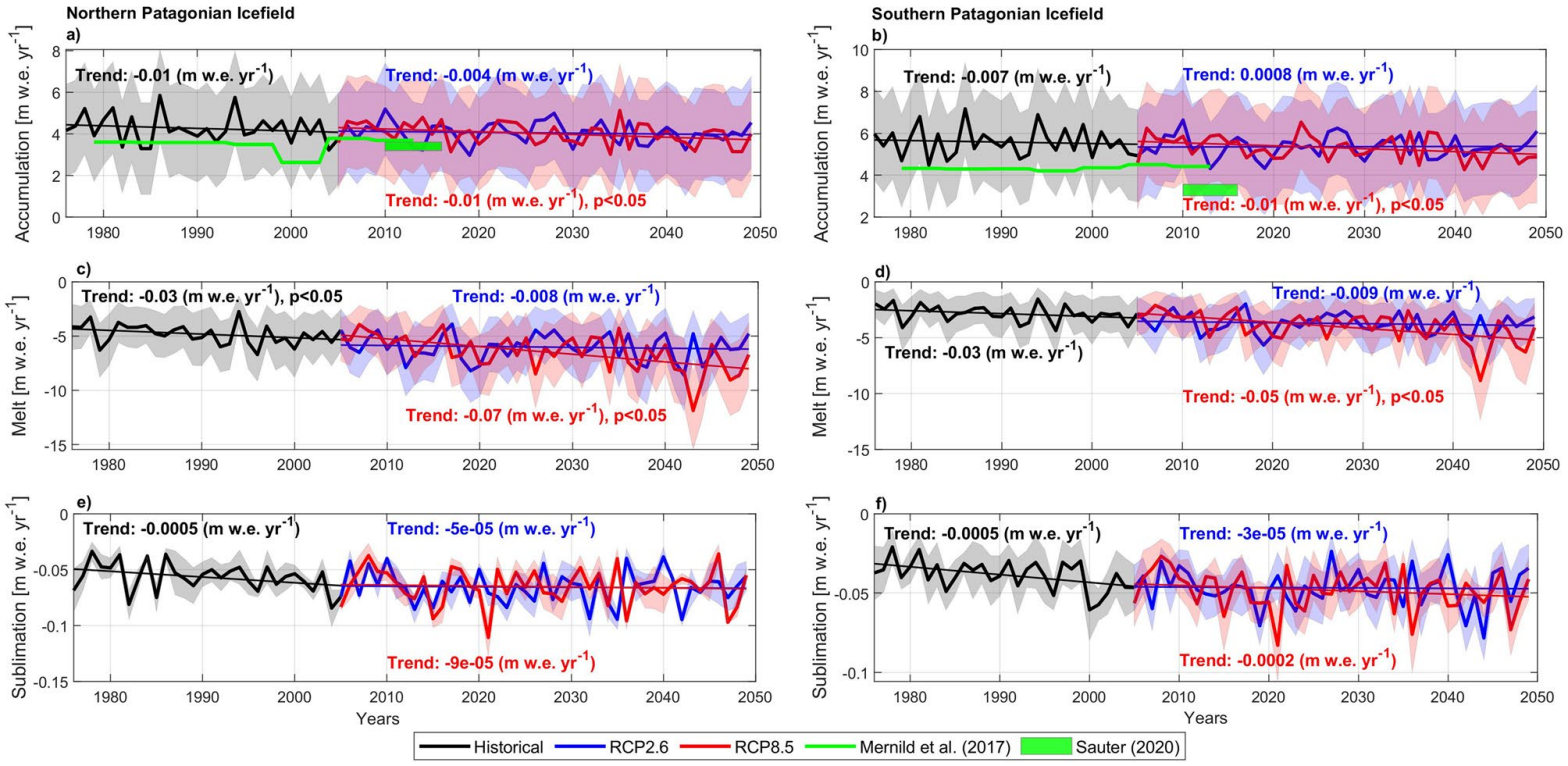
Annual values (hydrological years 1976/77–2049/50) and linear trends of the SMB components in m w.e. in NPI and SPI for the historical period and the two RCPs. (**a**,**c**,**e**) are accumulation, melt and sublimation in the NPI and (**b**,**d**,**f**) are accumulation, melt and sublimation in the SPI. Trends with the 95% confidence interval are shown. Significant trends are also indicated when p < 0.05. Trends in melt for RCP2.6 are significant where estimated between 1976 and 2050, reaching 0.03 ± 0.01 m w.e. in NPI and 0.02 ± 0.009 m w.e. in SPI.

Annual time series of the components of the SMB are given in Fig. 4. We found that the variance of the annual SMB depends largely on the ablation (R^2^ 0.88 and 0.94 for the NPI and SPI, respectively) rather than accumulation (R^2^ of 0.57 and 0.73, respectively).

Almost all the components in each scenario and during the historical period show trends that lead to negative SMB in the NPI and lower, but still positive, SMB in the SPI (Fig. 2). Surface accumulation in the NPI (Fig. 4a) showed an overall decrease for the historical period and both scenarios, being marginally statistically significant (p < 0.05) in the RCP8.5 scenario. SPI accumulation (Fig. 4b) showed a slight decrease during the historical period and RCP8.5 scenario and a slight increase for RCP2.6. Previous estimations of the snow accumulation trend for both icefields also showed a non-significant change at annual scale for the period 1980–2015; however a significant increase in snow accumulation during the autumn season was found for the same period^19^.

In the case of surface melt, an overall increase was determined over the historical period and both scenarios and for both icefields. The projected melt increase was statistically significant (p < 0.05) during the historical period in NPI (Fig. 4c) and for scenario RCP8.5 in both icefields. The magnitude of the change was larger in the NPI compared to SPI (Fig. 4d). Notably, the surface melt projected for the hydrological year 2043/44, reaches ~ 12 m w.e. yr^−1^ in NPI and ~ 9 m w.e. yr^−1^ in SPI. When excluding this large value, the trends were still significant at 0.008 m w.e. yr^−1^. Finally, sublimation showed an increase during the historical period and then plateaus during both future scenarios for both icefields, although its contribution to overall ablation is negligible according to our results.

### Ice loss and contribution to sea-level rise

In this section, we estimate the ice loss and the contribution to sea-level rise to year 2050 for both Patagonian Icefields. We make a key assumption that future rates of frontal ablation will be unchanged from those estimated during past and present conditions, masking the interannual variability as has been estimated, for instance, in Jorge Montt Glacier^31^. We calculate these frontal ablation rates by differencing our modelled SMB and previously published geodetic mass balances^3–12^ (Tables S2 and S3). Since the RCP simulations start in 2005, we used the mean SMB of the two scenarios in order to compare with the geodetic mass balances calculated after this year. For the NPI, for the period 1975–2016 (Table S2), we derive a mean frontal ablation contribution of 0.2 ± 0.9 m w.e. yr^−1^; for the SPI and between 1975 and 2016, the mean difference was 3.3 ± 0.8 m w.e. yr^−1^.

In terms of mass, for the NPI, we derive a total frontal ablation of 2.4 ± 3.6 Gt yr^−1^ in accordance with the robust estimation by Minowa et al.^41^ which estimated 2.5 ± 0.5 Gt yr^−1^. For the SPI, the estimated frontal ablation was 45.9 ± 15.9 Gt yr^−1^. This latter value is larger than the 40.7 Gt yr^−1^ estimated between 1975 and 2000 for the SPI, but lower than the 56.2 Gt yr^−1^ obtained between 2000 and 2011^25^. The frontal ablation estimation by Minowa et al.^41^ in the SPI reaches 21.6 ± 1.7 Gt yr^−1^, which is far lower than our estimation and previous ones. To match this value, the SMB in the SPI must decrease ~ 1.5 m w.e. Introducing this correction we obtained a mean SMB of 1.2 ± 1.6 m w.e. yr^−1^ for the historical period, while the projections changes to mean values of 0.1 ± 1.9 m w.e. yr^−1^ (RCP2.6) and − 0.3 ± 2.1 m w.e. yr^−1^ (RCP8.5).

**Figure 5:**
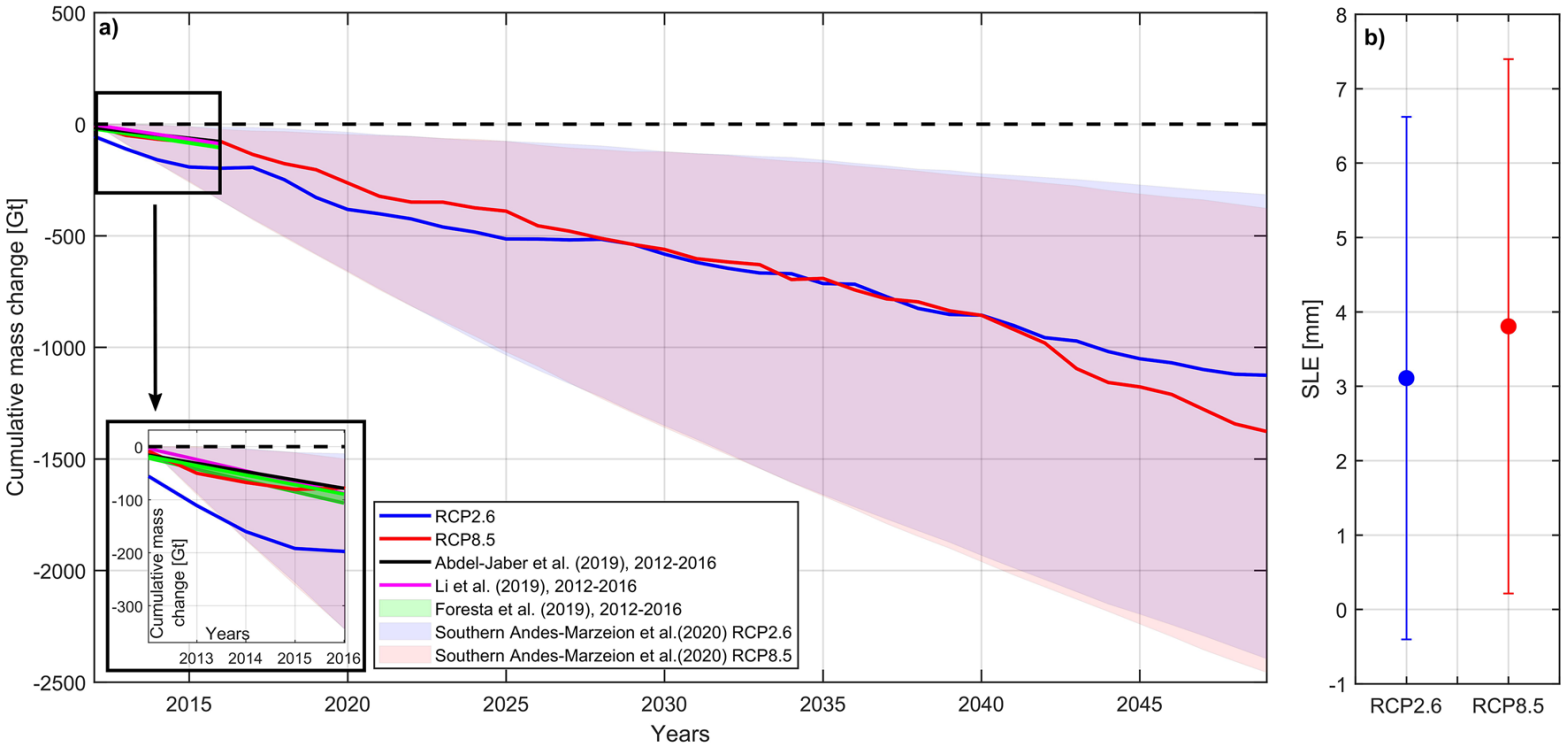
Cumulative ice loss estimated for both icefields combined between 2012 and 2050 (**a**) and total equivalent sea-level contribution to year 2050 (**b**). For comparison purposes, data from Marzeion et al.^43^ is showed (grey area). Marzeion et al.^43^ ice mass loss corresponds to all the models runs under scenarios RCP2.6 and RCP8.5 computed for the Southern Andes RGI region. Ice mass loss estimations by Abdel-Jaber et al.^12^, Foresta et al.^9^ and Li et al.^42^ for the four first years (2012–2016) are also shown. Inset, corresponds to a detailed view of this period.

Estimation of total ice mass loss between 2012 and 2050 and total sea-level rise equivalent to the year 2050 is shown in Fig. 5. Between 2012 and 2030 the greatest reduction in SMB is associated with the RCP2.6 scenario, after which the largest ice mass loss is projected by RCP8.5, particularly after 2040. RCP8.5 results agree well with those of Abdel-Jaber et al.^12^, Foresta et al.^9^ and Li et al.^42^ (Fig. 5) between 2012 and 2016, particularly so in the NPI where frontal ablation (and specifically calving) plays only a minor role. Our ice loss estimates are in the range of those shown by Marzeion et al.^43^ (Fig. 5), but it is worth noting that their results incorporate the entire Southern Andes, and with the exception of one model, neglect frontal ablation mechanisms entirely.

The total equivalent sea-level rise estimated here for both icefields, aggregated between 2012 and 2050 (Fig. 5b) reaches 3.1 mm (RCP2.6) and 3.8 mm (RCP8.5). Within this, the SPI contributes the largest part with 2.3 and 2.8 mm, respectively. The uncertainty, however, is large mainly due to the lack of knowledge in defining the actual range of the snow accumulation rate, which we introduce in our SMB modelling (Fig. 4a,b).

## Discussion

The results of our modelling showed that the surface melt increase during the period 1975–2050 is the main driver that leads to a reduction in the SMB in both Icefields. For the NPI, a negative SMB will prevails well into future decades. For the SPI, a correction was introduced in order to reconcile the SMB model results with the geodetic mass balances and the independent estimate of the frontal ablation^41^. In this case, mean SMB projected for the period 2005–2050, was barely positive (RCP2.6) or negative (RCP8.5). Moreover, a rise in the ELA throughout both icefields was projected. These changes agree with projected warming in the region (Fig. S2) and align with previous observations of the high sensitivity of Patagonian glaciers to ablation and air temperature variations^14^.

Qualitatively, an agreement between the surface and geodetic mass balance in the NPI seems to be related to the frontal characteristics of the glaciers. Many of them are located on land and terminate in small lakes, reducing the effect of frontal ablation. It has been estimated that calving flux was insignificant for the San Quintin Glacier, the largest glacier in the NPI, during the period 1975–2000^23^. The exception is San Rafael Glacier, which terminates in a lake connected to a fjord, where previous studies have defined the frontal ablation as being the most important ice loss mechanism^44^ being one order of magnitude higher than for most of the NPI glaciers^23^. Most glaciers in the SPI are tidewater-terminating in the west and freshwater-terminating in the east. Both fjords and lakes are of considerable depth, indicating frontal ablation plays an important role^31^. Nonetheless, the difficulties in the quantification of the frontal ablation lead to more simple assumptions, and thus, we assumed a constant value for the projections. As mentioned, Minowa et al.^41^ estimated a lower frontal ablation rate, which if we assume for the recent past and future conditions, generates a reduction in our SMB of 1.5 m w.e. This reduction is in the SMB uncertainty range (Fig. 2) and could be associated mainly with the high uncertainty in defining the actual snow accumulation (Fig. 4b). Indeed, the mean snow accumulation rate at the SPI after the correction, would be close to those previously estimated by Sauter^15^ who state that climate models tend to overestimate the precipitation in Patagonia. In case of implementing a single high-resolution regional climate model driven by a single global climate model, such biases can be amplified by combined sources of biases and uncertainties due to boundary conditions and regional climate model itself. In this respect, RegCM4.6 at 10 km resolution tends to overestimate the annual precipitation over the Andes cordillera in Patagonia if compared with gridded products derived from observations^35^. This overestimation is largely associated with the inherited errors from the boundary conditions as well as from the RegCM4.6 itself (e.g. physical configuration), and transferred to our snow accumulation estimate, which eventually leading to a high uncertainty, depending on the used method (Fig. 4b). The overestimation of the snow accumulation could be spatially concentrated on the western side, especially on the SPI, where lower ELA values were obtained in comparison to end-of summer snowline for particular years. Introducing the correction after the frontal ablation computed by Minowa et al.^41^, a rise in the ELA between 50 and 100 m was estimated, approaching the values previously observed (Table 1) although differences persist.

The overall reduction of the SMB projected for the next decades, will lead to a continuation or acceleration of the impacts associated with glacier shrinkage as has been observed in recent decades in the region, and also to an increase in the contribution to sea-level rise. Other than the positive or barely negative SMB that is projected for the SPI, a rise in ELA was projected for the NPI. The extent to which this impacts glacier length depends on several factors, including hypsometry^40^ and bed slope^45^. Previous work has shown that glaciers with a lower bed slope (e.g. San Quintin Glacier in NPI where negative SMB and ELA rise has been computed) could retreat around 50 m for each m of ELA rise^45^. This means that even without accounting for dynamic changes and mass loss via calving, some glaciers could retreat between 3 and 8 km within the next 30 years. It should be noted, however, that the projected rise in ELA in areas of the SPI could occur under the positive mass balance, meaning that glacier retreat in these zones solely associated with climate forcing will not occur. Potential glacier retreat will introduce new changes in the landscape. For instance, subglacial topography estimates suggest that pro-glacial lakes will likely form in several new areas of both the SPI and NPI^46^. These changes will in turn affect calving rates and subsequently glacier dynamics, making the prediction of future glacier evolution very complex^23^. Our estimation in this sense is, therefore, best considered first order. Actually, it is possible that in some areas frontal ablation rates could reduce as the glaciers thin, or even retreat out of their lacustrine or marine environments and onto land^23^. This scenario has already been proposed for the future evolution of Jorge Montt Glacier in the SPI; if the ongoing retreat continues, the glacier front will stabilise above the fjord level, reducing frontal ablation overall^31^.

Evidence found in the Patagonian Icefields, however, seems to suggest further positive feedbacks related to the increases in surface melt, and associated dynamic adjustments in calving glaciers^32^ and the appearance of supraglacial melt ponds^17^, which would serve to decrease the local surface albedo. Several studies^32,44^ have evidenced acceleration of glaciers in response to an increase of the basal sliding due to an input of meltwater percolating to the base. The temperate conditions of the ablation zones of Patagonian glaciers mean that refreezing would be minimal, and the subglacial hydrological network will likely play an important role in determining future dynamic behaviour. Under this assumption, glacier mass change could be not responding directly to the SMB change, but instead, it would depend importantly on one of the components of the SMB: the ablation and specifically the melt, whose increase due to higher air temperature, leads to a larger meltwater input to the glacier system. This hypothesis could explain in part the overall negative mass change detected in the SPI, while the SMB is positive or close to balance conditions.

**Figure 6:**
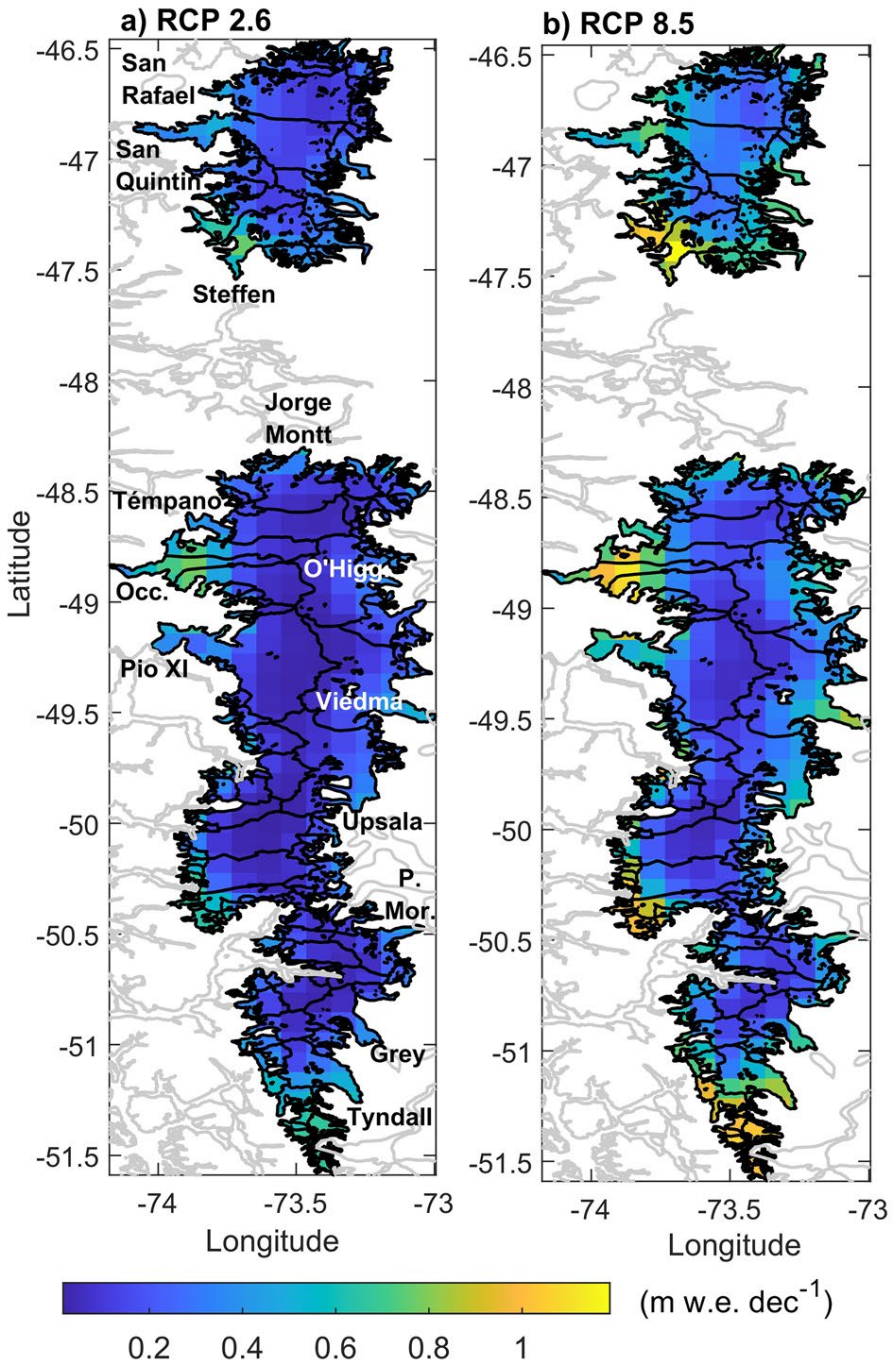
Spatially distributed melt trends over the Patagonian Icefields estimated with the EBM. Trends are from hydrological year 1976/77 to 2049/50, following after 2005 the pathway scenario RCP 2.6 (**a**) and the pathway scenario RCP8.5 (**b**). Black lines are the glacier basins and grey lines are coast lines. All the grid points are significant (p < 0.05).

Since glacier dynamics are closely connected to the climate conditions that define the input of meltwater to the system^32,33^, a qualitative projection and assessment of glacier response are possible with our results. The areas with the greatest projected positive trends in the surface melt, in addition to a strong thinning due to surface processes, are most likely to be affected by ice acceleration and increases in the calving flux^27^ and frontal ablation^33^. These areas are primarily located in the NE of the NPI (e.g. Steffen Glacier), along the western margin of the SPI (Occidental, Témpano, HPS-29 glaciers) and in the south of the SPI (Tyndall, HPS-38 glaciers) (Fig. 6). To a lesser extent, a future increase in surface melt was also estimated for the Upsala and Viedma glaciers in the SPI and San Quintin Glacier in the NPI (Fig. 6). In accordance with our results, most of these Patagonian glaciers are already thinning rapidly^7–9,12^. The only exception is Jorge Montt Glacier, located in the SPI. Here, along with other factors, glacier melt is also forced by warm water entering the fjord, explaining the strong retreat^31^ that is not suggested in our modelled increase in the surface melt (Fig. 6).

In terms of sea-level contribution, the total contribution projected to 2050 for both Icefields, represents a volume reduction between 22% (RCP2.6) and 27% (RCP8.5) if we compared it with the total sea-level equivalent of 14.1 mm estimated for both icefields^46^. The annual rates projected for both icefields are larger than those previously estimated from the LIA and most of the twentieth century (Table S4). For the 38 years period between 2012 and 2050, we estimated a mean annual rate of both scenarios of 0.067 mm yr^−1^ for the SPI and 0.024 mm yr^−1^ for the NPI, which in total represent 0.091 mm yr^−1^ for both icefields. These rates are larger than those estimated by Glasser et al.^13^ who estimated 0.0018 mm yr^−1^ between 1870 and 2010 in NPI and 0.005 mm yr^−1^ between 1750 and 2010 in SPI. Between 1945 and 1996^47^, the contribution of both icefields was estimated to be 0.032 mm yr^−1^ and between 1968/75–2000^3^ it was estimated as 0.042 mm yr^−1^. The rate of ice loss during most of the twentieth century is one order of magnitude higher than in previous centuries^13^. Additionally, in the last 6 years of the twentieth century, a new acceleration in ice mass loss was estimated^3^ reaching a sea-level contribution of 0.105 mm yr^−1^, while between 2002 and 2006^47^ the sea-level contribution of both icefields was estimated as 0.078 mm yr^−1^. Therefore, our projected sea-level contribution suggests that accelerated ice loss estimated for the end of 20th^3^ and the beginning of the 21st^42,48^ (see Table S4) centuries, will continue under both scenarios.

## Conclusions

In this work, we have quantified the present and future SMB for both the NPI and SPI using historical data and two Representative Concentration Pathways (RCP2.6 and RCP8.5) obtained from the RegCM4.6 model. Snow accumulation and ablation using an EBM were estimated, as well as their changes throughout the years. We estimated a reduction in the SMB for both icefields. For the NPI a mean reduction between 1.5 and 1.9 m w.e. was projected for the scenarios RCP2.6 and RCP8.5 respectively, while in SPI the reduction was within the range of 1.1 and 1.5 m w.e. Results of the modelled SMB and ELA in the SPI, suggests an overestimation of the accumulation estimated with the data from RegCM4.6. Overestimation of total precipitation in climate models for the Patagonia region has also been demonstrated in previous works^15,28^. The independent and robust computation of frontal ablation^41^ allowed correction of the modelled SMB for the SPI. Despite an overall reduction in SMB for the SPI, it was still projected to be positive (RCP2.6) or barely negative (RCP8.5) depending on the RCP. With the assumption that the annual rate of frontal ablation will remain unchanged, we estimated the contribution of the ice loss of the Patagonian Icefields to sea-level rise between 2012 and 2050, reaching between 3.1 mm (RCP2.6) and 3.8 mm (RCP8.5). The projected ELA rise indicates potential area reductions in the glaciers of the NPI as well as in some areas of the SPI, but limited to those with projected negative mass balances such as in the SW and only under the RCP8.5 scenario. The magnitude of ELA rise is spatially variable (between 26 and 118 m) and the impact at each glacier will depend on its topographic characteristics. The trend towards lower SMB overall is explained by an increase in surface melt and to a lesser extent by a reduction in snow accumulation. The input of infiltrating meltwater could act as a positive feedback not accounted for in our modelling, triggering ice-speed acceleration and ice loss due to an increase in frontal ablation of calving glaciers.

Although a complete quantification of future response depends on factors not accounted for by our modelling (e.g. ice dynamics), this knowledge of changes owing to climatic conditions gives new insights into recent trends of ice loss, their controls and uncertainties, and their likely impact on the future response of Patagonian glaciers through both space and time.

## Data and methods

### RegCM4.6 simulations

Regional Climate Model, version 4 (RegCM4.6) is a primitive equation and limited area model developed by the International Centre for Theoretical Physics^49^. The dynamical core of the RegCM4.6 is based on the hydrostatic version of the Penn State/NCAR mesoscale model MM5^50^. RegCM4.6 was used to downscale lateral boundary conditions derived from MPI-ESM-MR Earth System Model^34^ on a nested domain centralizing over Chile at 10-km spatial resolution for historical period (1976–2005) and projections (2006–2050) under the RCP2.6 and RCP8.5 scenarios^35^. This study makes use of daily mean near-surface temperature, total precipitation, wind speed, surface pressure, near-surface relative humidity, incoming shortwave and longwave radiation obtained from the downscaled RegCM4.6 simulations. RegCM4.6 simulations used in the present study are based on the land surface model Biosphere–Atmosphere Transfer Scheme of Dickinson et al.^51^ and the radiative scheme of the NCAR Community Climate System Model Version 3 (CCSM3)^52^. More information about the simulations, the performance of the RegCM4.6 as well as added value analysis are detailed in Bozkurt et al.^35^. Bozkurt et al.^35^ showed that high-resolution hindcast simulations (i.e. RegCM4.6 forced with reanalysis) robustly simulated the spatiotemporal variability of temperature and precipitation in southern Chile including the Patagonian Icefields, despite some discrepancies such as the overestimation of precipitation extremes. In addition, it was also highlighted that coarse resolution boundary conditions can miss important climate gradients imposed by complex topography in Chile. In this regard, downscaled simulations of RegCM4.6 forced with MPI-ESM-MR were found to exhibit an added value for both temperature and precipitation by resolving local-scale features in a large part of Chile, including grid points in the Patagonian Icefields. Nonetheless, there still exist some persistent uncertainties in the RegCM4.6 forced with MPI-ESM-MR such as precipitation overestimation over the Andes cordillera in Patagonia. In terms of the hypsometry, the topography of RegCM4.6 (interpolated from GTOPO30) represents quite well the lower elevations and the plateau zone, although underestimation in the higher elevations is evident compared with the Shuttle Radar Topography Mission (SRTM) at 1 km resolution (Fig. S1). The areas better represented by the RegCM4.6 topography are between 65 to 70% of the total area in each icefield. Over ~ 1500 m a.s.l. the representation is not good as a clear underestimation of the elevation exists. In terms of melt, a minimal impact is expected considering that most of the melt occurs at lower elevations, but some underestimation of the sublimation is likely.

### Surface mass balance

A surface mass balance (SMB) was computed for both icefields. The components of the SMB were surface melt, sublimation and accumulation and were estimated for each RegCM4.6 grid point that intersects the icefields. Glacier wide-surface mass balance and its components correspond to the mean values of each icefield. Even though the resolution of the input data is coarse (~ 10 km), we showed some results distributed by sub-zones defined in Fig. 1. Considering the resolution of the RegCM4.6 as well as the SMB, the results are more representative of the large glaciers. More high resolution modelling studies combining both dynamical and statistical downscaling/bias-correction techniques might be useful to overcome local-scale biases that are associated with the complex topography.

### Ablation: energy balance modelling

A distributed energy balance model (EBM) was applied using daily data obtained from the RegCM4.6 regional model between 1 April 1976 and 31 March 2050, the Southern hemisphere mid-latitudes hydrological year. The details of the equations are given in the Supplementary Material and correspond to the bulk method to estimate sensible, latent and rainfall fluxes including stability corrections in the first two fluxes. In addition to the meteorological data obtained from RegCM4.6 (previous section), we estimated additional variables necessary to feed the EBM. These are surface temperature, air and surface vapour pressure, outgoing longwave radiation, surface albedo and surface roughness. The first three variables, were estimated with standard equations and parametrization and using the data from RegCM4.6. Details of these equations and parametrization are given in the Supplementary Material. Outgoing longwave radiation was in turn estimated using the distributed field of the surface temperature and assuming a surface emissivity equal to 1 in the Stefan-Boltzmann law. The surface albedo was estimated following Oerlemans and Knapp^53^. Surface roughness was estimated using the surface albedo values as a proxy. Here, albedo values between 0.35 to 0.60 were associated with ice and firn (higher surface roughness) and between 0.60 to 0.80 to different snow ages (lower surface roughness). From the EBM we obtained the melt and the sublimation at the daily time step, which is then calculated at the annual time step. The use of daily data allows a better representation of the inter-daily variability of the albedo and the impact in the reflected shortwave radiation. In order to quantify the uncertainty in one of the parametrizations, we run two experiments using different magnitudes of the glacier cooling effect assuming − 1 °C and − 3 °C, respectively. These values were taken from the analysis of air temperature data observed on-glacier and compared with data measured off-glacier at both sides of the SPI^18^. Although there are other inherent uncertainties in the modelling that are hard to quantify, such as the parametrization of the surface roughness, the use of an EBM makes it possible to integrate more meteorological variables than a temperature-index model allowing a more complete evaluation of the impacts of the projected climate change over glaciated areas.

### Snow accumulation

We estimated the snow accumulation over the glacier surface following the method of Weidemann et al.^28^ and Schaefer et al.^23,25^ previously applied at single glaciers and at the Patagonian Icefield scale, respectively. This method showed the closest magnitude representation compared with short-term snow accumulation in the SPI^19^. We repeat this exercise using the RegCM4.6 data from each RCP. This is presented in Figure S3, where a clear overestimation is detected using the RCP8.5 data, while RCP2.6 shows also an overestimation along the period but a total accumulation close to the estimated accumulation derived from the ultrasonic-depth gauge (UDG). The source of the overestimation seems to be related to the climate model (MPI-ESM-MR Earth System Model) used to forced RegCM4.6 due to the fact that snow accumulation estimates of RegCM4.6 forced by ERA-Interim show a range of value closest to the observed by the UDG in a previous study^19^. However, as the uncertainty in precipitation is high^15^ and hence also in the snow accumulation, we quantify the uncertainty based on different methods to estimate the partitioning between rain and snow. These methods are described in full in Bravo et al.^19^. The input for these estimations was total precipitation, air temperature and relative humidity to estimate dew point temperature for one of the methods^19^. The daily snow accumulation was then used to estimate annual accumulation at each hydrological year.

### Equilibrium-line altitude

Equilibrium-line altitudes (ELA) by zone (Fig. 1b,c) were estimated using the mean value of the specific SMB by elevation range and for each hydrological year. The ELA was then estimated at the elevation where the annual SMB was equal to 0 m.w.e. In order to compare with previous ELA estimations, annual values for some years were extracted (Table 1), while the differences between the historical period and each RCP was estimated based on the long-term mean value.

### Ice loss estimation and contribution to sea-level rise

The ice loss and contribution to sea-level rise were estimated using previous area and volume data. For the first case, we assume as initial condition the area estimated for the 2011 year by Davies and Glasser^54^ and the total volume estimated by Millan et al.^55^ which was computed using airborne gravity and radar measurements taken between 2012 and 2016 and complemented with ice thickness, modelled by Carrivick et al.^46^. The areas used were 13,219 km^2^ for the SPI and 3976 km^2^ for the NPI. Volumes were 3632 km^3^ in the SPI and 1124 km^3^ in the NPI. Using these data, we estimated the factors for a bulk volume-area scaling at each icefield. For glacier area *S* the volume *V* was estimated as *V* = *cS*^*γ*^^56^. The empirical factor *c* was estimated, finding that the best adjustment was obtained with *c* equal to 0.01264 in NPI and 0.007825 in SPI. The exponent parameter *γ* is constant and its value is 1.375. The main assumption to estimate total ice loss in the future was that the annual rate of frontal ablation in the SPI is equal to the present conditions. The rate of present frontal ablation was estimated following Schaefer et al.^25^ and assumed that the differences between geodetic mass balance and modelled SMB correspond to the frontal ablation. In our case, we estimate the mean of the differences of the values as shown in Tables S2 and S3.

Using the initial conditions, the modelled SMB plus frontal ablation (in the case of the SPI) and the volume-area scaling parameters, the volume loss at the yearly time step was estimated (${\Delta V}_{y}$) and consecutively added to the volume of the previous year (${V}_{y}$). The new volume, and using the defined parameters ($c$ and *γ*) in turn is used to estimate the new area for the following year (${A}_{y+1}$, which is then multiplied with the SMB). This relationship takes the following form^56^:

$${A}_{y+1}={\left(\frac{{V}_{y}+{\Delta V}_{y}}{c}\right)}^{\frac{1}{\gamma }}$$

The volume loss is then adjusted by the area in each time step. Finally, volume is converted to mass using an ice density of 900 kg m^−3^, and the resulting ice loss (Gt) is converted to sea-level equivalent.

## Supplementary Information﻿

Supplementary Information.
