# Supplementary material for: Projected increases in surface melt and ice loss for the Northern and Southern Patagonian Icefields
Source: Sci Rep. 2021 Aug 19;11:16847. doi: 10.1038/s41598-021-95725-w (PMC8376928; doi:10.1038/s41598-021-95725-w)
Supplement: Supplementary file 1 — Supplementary Information. [file 41598_2021_95725_MOESM1_ESM.pdf]

Supporting Information for

Projected increases in surface melt and ice loss for the Northern and Southern Patagonian Icefields

Claudio Bravo[1,2], Deniz Bozkurt[3], Andrew N. Ross[4] and Duncan J. Quincey[2]

[1] Centro de Estudios Científicos (CECs), Valdivia, Chile.

[2] School of Geography, University of Leeds, Leeds, United Kingdom.

[3] Department of Meteorology, University of Valparaíso, Valparaíso, Chile and Center for Climate and Resilience Research (CR)2, Santiago, Chile.

[4] School of Earth and Environment, University of Leeds, Leeds, United Kingdom.

Contents of this File

- Text and equations of Energy Balance Modelling
- Figure S1 to S3
- Table S1 to S3

Energy Balance Modelling

An energy balance (EB) model was applied using RegCM4.6 data. Computation of the energy available for melting, Q_m (W m^{-2}) was made at each pixel following the equation:

$$Q_m = (1-\alpha)S_{in} + L_{in} + L_{out} + Q_h + Q_l + Q_r, \qquad (1)$$

where S_{in} and α are incoming shortwave radiation and the albedo, L_{in} and L_{out} are incoming and outgoing longwave radiation and Q_h and Q_l are the turbulent fluxes of sensible and latent heat, respectively. Q_r is the sensible heat brought to the surface by rain. The conductive heat flux was considered negligible due to the predominantly positive air temperatures and the temperate conditions of the glacier surface[40]. S_{in} and L_{in} were obtained directly from the RegCM4.6.simulations.

The albedo α, was obtained following Oerlemans and Knapp[53] using the relationship:

$$\alpha_s^t = \alpha_{fi} + \left(\alpha_{fr} - \alpha_{fi}\right) \cdot e^{-\Delta t/t^*}, \qquad (2)$$

$$\alpha^t = \alpha_s^t + (\alpha_{ice} - \alpha_s^t) \cdot e^{-d/d^*}, \qquad (3)$$

where α^t corresponds to the global albedo at the surface on a specific t day. α_s^t corresponds to the snow albedo at the surface on a t day. The α_{fr} and α_{fi} parameters are related to fresh snow albedo (0.85) and firn or old snow albedo (0.53), respectively. α_{ice} represents a specific glacier ice albedo (0.35), while t^*corresponds to the time scales that represent the transition of fresh snow albedo to firn (3 days). The Δt term refers to days since the last snowfall event. The d and d^* parameters correspond to the snow depth (in meters), and scale coefficient of snow depth (0.032 m), respectively.

The outgoing longwave radiation (L_{out}) was calculated using the surface temperature (T_s) and assuming a surface emissivity equal to 1 in the Stefan-Boltzmann law.

$$L_{out} = \varepsilon_{sur} \cdot \sigma T_s{}^4, \quad (4)$$

To estimate the surface temperature (T_s) we used as a proxy the dew point temperature (T_d) for the snow surface and the assumption of constant surface temperature (0°C) under melt conditions on glacier surfaces, namely when the air temperature is positive[57]. The T_d approach calculated at a standard height is a reasonable first-order approximation of daily T_s[58] and was calculated using the Magnus-Teten approach[58,59]:

$$T_s = T_d = \frac{c\left[ln(f)+\frac{bT_a}{c+T_a}\right]}{b-ln(f)-\frac{bT_a}{c+T_a}}, \quad (5)$$

As the aim was to estimate snow surface temperature when T_a<0°C, the coefficients used were b= 22.587 and c= 273.86°C[58].

The turbulent sensible heat fluxes were calculated using the bulk approach[60]. In the case of the sensible heat flux;

$$Q_h = \rho_a c_a C^* u[T_a - T_s]\,(\Phi_m \Phi_h)^{-1}, \quad (6)$$

where u is wind speed in m s^{-1}, T_a is air temperature in *K* and T_s is glacier surface temperature. $C^*$is a dimensionless transfer coefficient, which is a function of the surface aerodynamic roughness (z_o):

$$C^* = \frac{k^2}{ln^2\left(\frac{z}{z_o}\right)}, \quad (7)$$

where z is the height above the surface of the *T* and u measurements (2 m) and k is the von Kárman's constant (0.4). Due to the absence of microtopographic measurements, z_o was prescribed according to the albedo using values taken from Brock et al.[61] (Table S1). It has been suggested that surface roughness also affects surface albedo, especially on snow surfaces[62]. ρ_a is the density of air, which depends on atmospheric pressure *P* (in Pa):

$$\rho_a = \rho_a^o \frac{P}{P_0}, \quad (8)$$

where ρ_a^o (1.29 kg m^{-3}) is the density at standard pressure P_0 (101300 Pa). Finally, c_a is the specific heat of air at a constant pressure (J kg^{-1} K^{-1}) calculated as[62]:

$$c_a = 1004.67\left(1 + 0.84\left(0.622\left(\frac{e}{P}\right)\right)\right), \quad (9)$$

The latent heat flux Q_l is:

$$Q_l = \frac{0.622\rho_a L_{v/s} C^* u[e_a - e_s]}{P}\,(\Phi_m \Phi_h)^{-1}, \quad (10)$$

where e_a is air vapour pressure, e_s is the vapour pressure at the glacier surface which was assumed saturated and hence depends only on surface temperature[63]. $L_{v/s}$ is the latent heat of vaporization or sublimation, depending on whether the surface temperature is at melting point (0°C) or below the melting point (<0°C), respectively.

Stability corrections were applied to turbulent fluxes using the bulk Richardson number (Ri_b), which is used to describe the stability of the surface layer[64]:

for Ri_b positive (stable): $(\Phi_m \Phi_h)^{-1} = (\Phi_m \Phi_v)^{-1}$

$$=(1-5Ri_b)^2, \qquad (11)$$

for Ri_b negative (unstable): $(\Phi_m \Phi_h)^{-1} \;\; = (\Phi_m \Phi_v)^{-1}$

$$= (1-16Ri_b)^{0.75}. \qquad (12)$$

Ri_b is used to describe the stability of the surface layer:

$$Ri_b = \frac{g(T-T_s)(z-z_0)}{Tu^2}, \qquad (13)$$

where g is the acceleration due to gravity.

The rain heat flux (Q_r) is a function of the rainfall rate intensity (R, m s^{-1}) and the rain temperature (T_r) is assumed to be equal to the air temperature[65]:

$$Q_r = \rho_w c_w R[T_r - T_s], \qquad (14)$$

where p_w is the density of water and c_w is the specific heat of water (4180 J kg^{-1} K^{-1}). The rainfall intensity was obtained from the total precipitation RegCM4.6 dataset at a daily time step. The rainfall was obtained from the total precipitation using the Phase Partitioning Methods (PPM) explained in the Snow Accumulation Methods section.

Melt was assumed to occur only when the glacier surface was at 0°C and Q_m was positive. The melt rate (M) was calculated using:

$$M = \frac{Q_m}{L_m \rho_w}, \qquad (15)$$

where L_m is the latent heat of fusion and ρ_w is the water density (1000 kg m^{-3}).

The sublimation rate (S) was calculated as[60]:

$$S = \frac{Q_l}{L_s \rho_w}, \qquad (16)$$

where L_s is the latent heat of sublimation. We assume that negative latent heat fluxes (Q_l) correspond only to sublimation[66].

References (not listed in the main manuscript)

57 Oerlemans, J., & Klok, E.J. Energy balance of a glacier surface: analysis of Automatic Weather Station data from the Morteratschgletscher, Switzerland. *Arct. Antarct. Alp. Res.* **34**(4), 477-485 (2002).

58 Raleigh, M.S., Landry, C.C., Hayashi, M., Quinton, W.L., & Lundquist, J.D. Approximating snow surface temperature from standard temperature and humidity data: new possibilities for snow model and remote sensing evaluation. *Water Resour. Res.* **49**(12), 8053–8069. 101002/2013WR013958 (2013).

59 Murray, F.W. On the computation of saturation vapor pressure. *J. Appl. Meteorol.* **6** (1), 203–204 (1967).

60 Cuffey, K.M., & Paterson, W.S.B. *The physics of glaciers*, 4th edn. Butterworth-Heinemann, Oxford (2010).

61 Brock, B.W., Willis, I.C. & Sharp, M.J. Measurement and parameterization of aerodynamic roughness length variations at Haut Glacier D'Arolla, Switzerland. *Ann. Glaciol.* **52**(177), 281–297; 10.3189/172756506781828746 (2006).

62 Manninen, T., Lahtinen, P., Anttila, K., & Riihelä, A. Detection of snow surface roughness and hoar at Summit, Greenland, using RADARSAT data. *Int. J. Remote Sens.* **37**(12), 2860-2880; 10.1080/01431161.2015.1131873 (2016).

63 Brock, B., & Arnold, N. A spreadsheet-based (Microsoft Excel) point surface energy balance model for glacier and snowmelt studies. *Earth Surf. Proc. Land.* **25**(6), 649–658 (2000).

64 Oke, T.R. *Boundary Layer Climate*, 2nd ed., Methuen, London (1987).

65 Hock, R., & Holmgren, B. A distributed energy balance model for complex topography and its application to Storglaciären, Sweden. *J. Glaciol*. **51**(172), 25-36 (2005).

66 Ayala, A., Pellicciotti, F., Peleg, N., & Burlando, P. Melt and surface sublimation across a glacier in a dry environment: distributed energy-balance modelling of Juncal Norte Glacier, Chile. *J. Glaciol*. **63** (241), 803–822 (2017).

Figures and Tables

Figure S1. Hypsometry curves of each icefield constructed with RegCM4.6 topography (10 km resolution) and the Shuttle Radar Topography Mission (SRTM) at 1 km resolution. a) curves for the Northern Patagonian Icefiled (NPI) and b) curves for the Southern Patagonian Icefiled (SPI).

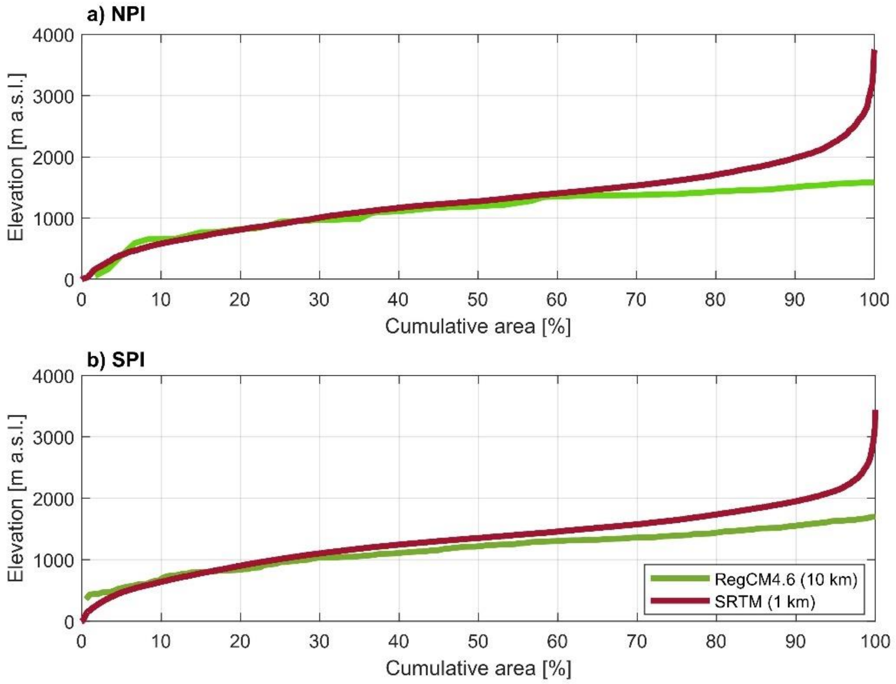

Figure S2. Annual air temperature anomalies over NPI (a) and SPI (b) for both scenarios (RCP2.6 and 8.5) estimated with respect to the mean of the historical period (1976-2004).

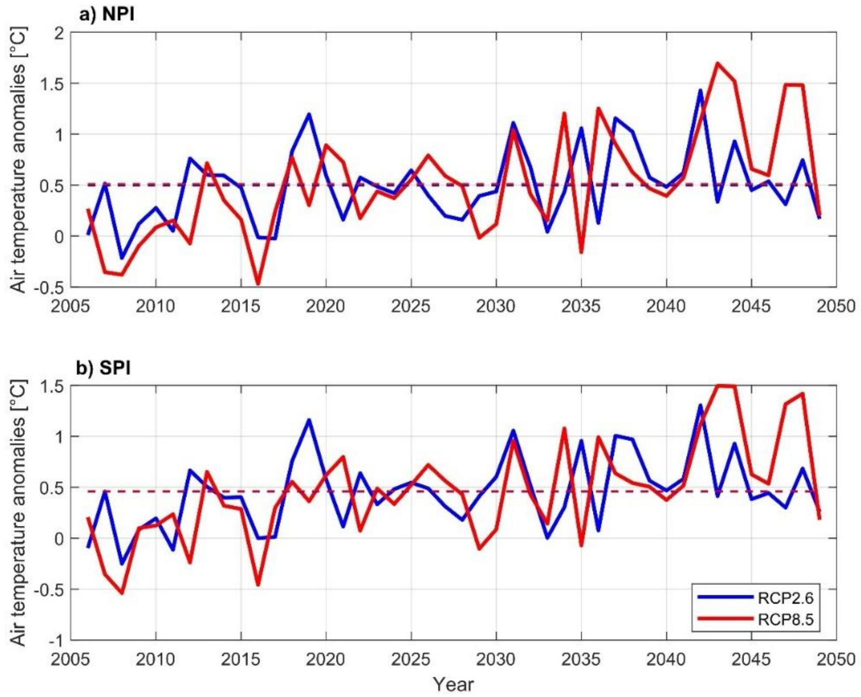

Figure S3. Snow accumulation estimated using precipitation data from the MPI-ESM-MR RegCM4.6 scenarios RCP2.6 (blue line) and RCP8.5 (red line), range of accumulation for the same period and grid point estimated by Bravo et al.[19] using ERA-Interim RegCM4.6 (grey area) and snow accumulation derived from an ultrasonic-depth gauge (UDG, model SR50) installed during three months (October to December, 2015) in the plateau of the Southern Patagonian Icefield (black line, 48°50'02"S/ 73°34'51"W/ 1415 m a.s.l.).

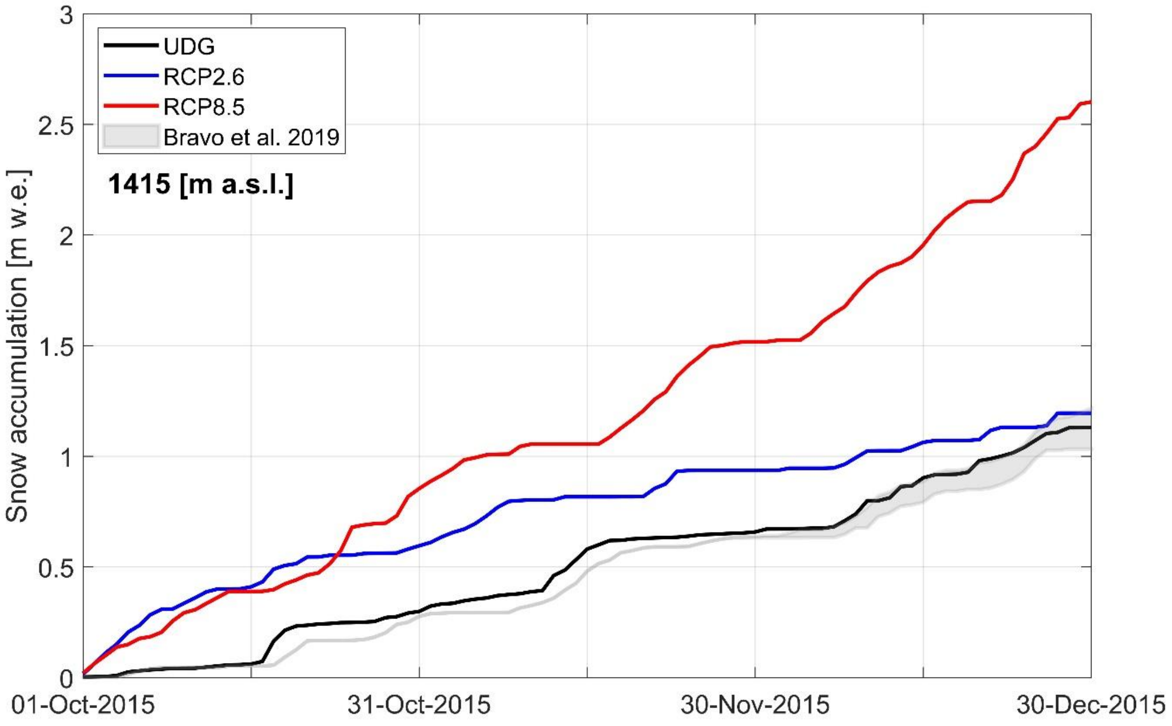

Table S1. Fixed surface roughness for albedo range depending on the surface type.

Surface type	Albedo	Roughness (m)
Ice	0.35-0.45	0.01
Firn	0.45-0.60	0.007
Old snow	0.60-0.70	0.005
Clean new snow	0.70-0.80	0.001
Fresh snow	>0.80	0.0002

Table S2. Comparison of geodetic mass balance and modelled surface mass balance estimated in this work in the NPI. For those period started in 2000/01, we used values of the historical period until 2004 and then to complete the comparison period, we used the values for each scenario.

**Period**	**Reference**	**Geodetic mass balance (m w.e.)**	**This work (m w.e.)**	
1975-2000	Rignot et al.[3]	-0.69±0.09	-0.49±2.13	
1980-2002	Rivera et al.[4]	-1.53±0.82	-0.71±2.17	
			RCP2.6	RCP8.5
2001-2011	Willis et al.[6]	-0.96±0.04	-1.14±2.17	-0.84±2.14
2000-2012	Dusaillant et al.[8]	-1.06±0.15	-1.22±2.23	-1.07±2.21
2000-2012	Abdel-Jaber et al.[12]	-0.91±0.04	-1.57±2.30	-1.14±2.25
2000-2014	Braun et al.[10]	-0.85±0.07		
2000-2015	Dusaillant et al. [11]	-0.98±0.27	-1.70±2.31	-1.14±2.25
2012-2016	Abdel-Jaber et al.[12]	-1.22±0.16	-3.29±2.64	-1.35±2.40
2011-2016	Foresta et al. [9]	-1.58±0.27	-2.38±2.53	-1.08±2.33

Table S3. Comparison of geodetic mass balance and modelled surface mass balance estimated in this work in the SPI. For those period started in 2000/01, we used values of the historical period until 2004 and then to complete the comparison period, we used the values for each scenario.

<table>
<tr><th rowspan="2">Period</th><th rowspan="2">Reference</th><th rowspan="2">Geodetic mass balance (m w.e.)</th><th colspan="2">This work (m w.e.)</th></tr>
<tr></tr>
<tr><td>1975-2000</td><td>Rignot et al.[3]</td><td>-0.88±0.05</td><td colspan="2">2.83±1.57</td></tr>
<tr><td>1995-2000</td><td>Rignot et al.[3]</td><td>-2.21±0.29</td><td colspan="2">2.70±1.68</td></tr>
<tr><td></td><td></td><td></td><td>RCP2.6</td><td>RCP8.5</td></tr>
<tr><td>2000-2012</td><td>Willis et al.[5]</td><td>-1.49±0.09</td><td rowspan="3">2.22±1.72</td><td rowspan="3">2.39±1.68</td></tr>
<tr><td>2000-2012</td><td>Braun et al.[10]</td><td>-0.86±0.08</td></tr>
<tr><td>2000-2012</td><td>Abdel-Jaber et al.[12]</td><td>-0.97±0.03</td></tr>
<tr><td>2000-2015</td><td>Malz et al.[7]</td><td>-0.89±0.24</td><td rowspan="2">1.83±1.82</td><td rowspan="2">2.31±1.74</td></tr>
<tr><td>2000-2015</td><td>Dusaillant et al.[11]</td><td>-0.96±0.29</td></tr>
<tr><td>2012-2016</td><td>Abdel-Jaber et al.[12]</td><td>-0.79±0.13</td><td>0.59±2.18</td><td>2.15±1.91</td></tr>
<tr><td>2011-2016</td><td>Foresta et al.[9]</td><td>-0.98±0.11</td><td>0.96±2.07</td><td>2.09±1.89</td></tr>
</table>

Table S4. Comparison of estimated sea-level contribution from the Southern Andes[2] and the Patagonian Icefields[3,13,23,41,47,48]. First rows are the total contribution for the respective periods, while the rest of the rows show the annual rate estimate for the respective period.

	**Southern Andes (RGI region)**		
Zemp et al. (2019)[2]	3.3 mm		1961-2016
	**NPI and SPI**		
This work	3.1 - 3.8 mm		2012-2050
	**NPI**	**SPI**	
Glasser et al. (2013)[13]	0.0018 mm yr^{-1}	0.005 mm yr^{-1}	LIA-2010
Rivera et al. (2002)[47]	0.032 mm yr^{-1}		1945-1996
Rignot et al. (2003)[3]	0.042 mm yr^{-1}		1968/75-2000
Rignot et al. (2003)[3]	0.105 mm yr^{-1}		1995-2000
Chen et al. (2007)[48]	0.078 mm yr^{-1}		2002-2006
Li et al. (2019)[43]	0.066 mm yr^{-1}		2002-2016
Schaefer et al. (2013)[23]	0.016 mm yr^{-1}		21st Century
This work	0.091 mm yr^{-1}		2012-2050
	0.024 mm yr^{-1}	0.067 mm yr^{-1}	
